# DAG-CTFL: DAG Blockchain Cross-Layer Authentication Framework for Trustworthy IoV Federated Learning

**DOI:** 10.3390/e28060589

**Published:** 2026-05-26

**Authors:** Longxia Liao, Long Chen

**Affiliations:** 1 School of Information and Software Engineering, East China Jiaotong University, Nanchang 330013, China; 2 School of Electrical and Automation Engineering, East China Jiaotong University, Nanchang 330013, China; tyrone28@126.com

**Keywords:** internet of vehicles, privacy-preserving federated learning, cross-layer collaborative authentication, DAG blockchain, dynamic trust management, differential privacy

## Abstract

Privacy-preserving federated learning in the internet of vehicles (IoV) requires low-latency authentication, bounded privacy leakage, and robustness against malicious model updates. However, most existing studies separately design communication authentication and federated learning protection, which leads to duplicated overhead and weak resistance to cross-layer attacks. To address this issue, this paper proposes a DAG blockchain-enabled cross-layer authentication framework for trustworthy IoV federated learning (DAG-CTFL). The framework reuses authentication operations across V2X message verification and model-update delivery, incorporates trust-aware batch verification, and organizes cross-layer evidence through a two-tier DAG blockchain. In addition, differential privacy is used to reduce information leakage from uploaded model updates, while cross-layer trust evaluation improves resilience against poisoning and forged-identity attacks. Experimental results on MNIST and CIFAR-10 show that DAG-CTFL reduces single-message verification overhead by 8.2–56.1%, lowers batch-verification latency by 19.2–56.4%, and maintains model accuracy above 85% under 15% malicious nodes. These results demonstrate that DAG-CTFL achieves an effective balance among privacy preservation, authentication efficiency, and cross-layer robustness in IoV federated learning.

## 1. Introduction

The maturity of 5G-V2X communication technologies has made IoV a core component of next-generation intelligent transportation systems [1]. In this environment, many vehicular services rely on dense and time-sensitive message exchange among vehicles, roadside units (RSUs), and edge servers. Therefore, communication efficiency and authentication latency are not secondary concerns; they directly affect whether upper-layer intelligent services can be deployed at scale.

Privacy-preserving federated learning (PPFL) has become an attractive application-layer solution because it allows distributed model training without exposing raw local data [2]. In IoV, however, PPFL is meaningful only when the underlying communication process is trustworthy, lightweight, and resistant to realistic adversaries. If V2X message authentication and model-update protection are implemented through two separate security pipelines, the system incurs repeated cryptographic cost, redundant signaling overhead, and fragmented trust evaluation.

This coupling makes IoV federated learning fundamentally a cross-layer communication-security problem rather than a standalone learning problem. Open wireless links remain vulnerable to identity forgery, message tampering, replay, and impersonation attacks. At the same time, malicious participants may inject poisoned model updates, manipulate local training, or exploit side information to infer private training data at the application layer. Recent cryptanalysis of a chaos- and S-box-based video cryptosystem further shows that design-level weaknesses in multimedia encryption, such as insufficient key-dependent diffusion, may expose protected visual data and undermine the assumed security of data transmission [3]. In IoV federated learning, such risks are further amplified by the massive exchange of sensory and multimedia data. In highly dynamic vehicular scenarios, these threats reinforce each other and evolve into cross-layer compound attacks [4,5].

However, centralized aggregation creates a single-point bottleneck, and conventional chain-based blockchains struggle to meet the low-latency and high-concurrency demands of dense vehicular networks. As a result, a practical PPFL framework for IoV must simultaneously provide robust privacy guarantees under realistic adversaries, low-latency source authentication, efficient batch verification, bounded communication overhead, and deployability on resource-constrained vehicles and RSUs [4,6].

Prior studies usually optimize only one side of this problem. Communication-layer designs such as TABMA [7] mainly emphasize fast verification and malicious-message filtering, whereas DAG-FL [8] and related methods [9,10] focus on FL robustness against poisoning or Sybil-style manipulation. Existing DAG blockchain designs also mainly support a single service process rather than communication-learning trust fusion [11]. Consequently, current solutions still suffer from protocol redundancy, repeated cryptographic cost, weak cross-layer coupling, and limited resistance to compound attacks.

To address these limitations, this paper proposes DAG-CTFL, a DAG blockchain-enabled cross-layer authentication framework for trustworthy IoV federated learning. DAG-CTFL places privacy-preserving learning and communication efficiency at the center of the design by reusing authentication operations across V2X messaging and model-update delivery, introducing trust-aware batch verification, and organizing cross-layer evidence through a scalable two-tier DAG blockchain. Federated learning is treated as the application-layer service scenario in which these communication and trust mechanisms operate. The main contributions are as follows:

1. We propose a cross-layer authentication framework for trustworthy IoV federated learning (DAG-CTFL). It achieves a strict closed-loop trust coupling by reusing the same signature system for both low-level V2X message verification and high-level model-update authentication. This fundamental design deeply couples communication security with federated learning protection, eliminating the redundant overhead of dual-independent protocols.
2. We design a scalable two-tier DAG blockchain architecture for multi-level evidence management. To address the scalability bottlenecks in large-scale urban IoV, the local tier utilizes a high-concurrency Tangle for rapid vehicular interactions, while the global tier employs a trust-weighted PBFT committee mechanism. This limits the communication complexity to $O(C^{2})$, ensuring efficient and low-latency cross-regional state synchronization.
3. We develop a trust-privacy collaborative defense mechanism and provide extensive experimental validation. By synergizing cross-layer trust evaluation with differential privacy, the framework dynamically filters malicious model updates. Comprehensive evaluations on MNIST and CIFAR-10 datasets demonstrate that DAG-CTFL significantly reduces batch verification latency and maintains high model utility (e.g., preventing accuracy collapse) even under high-intensity composite attacks.

The rest of this paper is organized as follows: Section 2 reviews the related work and limitations of existing studies. Section 3 defines the system model and threat model. Section 4 elaborates the core design of the DAG-CTFL framework. Section 5 presents the security analysis and proofs of the framework. Section 6 verifies the performance and robustness of the framework through simulation experiments. Section 7 concludes the paper and discusses future research directions.

## 2. Related Work

This section briefly reviews three related directions: IoV identity authentication, secure federated learning in IoV, and blockchain-enabled trust management, and then highlights how DAG-CTFL differs from representative prior studies.

### 2.1. IoV Identity Authentication Technologies

Identity authentication is the basis of communication security in IoV, where existing studies mainly pursue low overhead, privacy preservation, and high-concurrency verification. PKI-based schemes suffer from certificate-management overhead in dynamic vehicular environments [12], whereas IBC removes certificates at the cost of key-escrow risk [13]. CL-PKC therefore becomes a practical direction because it avoids both limitations while remaining lightweight for vehicular deployment [14].

To improve verification efficiency, many schemes introduce batch authentication. Representative studies in [15,16] reduce verification cost, but they remain sensitive to invalid messages and may incur extra delay during re-verification. Blockchain-assisted schemes such as BATS [17], BEBA [18], and our previous TABMA scheme [7] further combine trust or anchoring mechanisms to improve robustness and traceability. However, these studies still target the communication layer only and do not evaluate the reliability of FL participation or model contribution.

### 2.2. Security of Federated Learning in IoV

Federated learning in IoV mainly faces privacy leakage and model poisoning threats [19]. Differential privacy and homomorphic encryption are common privacy-preserving solutions, but they either degrade model utility when noise is aggressive [20,21] or introduce high computation cost for resource-constrained vehicles [22]. For robustness, trust-guided filtering and blockchain-assisted traceability have also been adopted to detect malicious updates. Representative studies such as [8,9,23,24] improve aggregation reliability through reputation management, DAG-based orchestration, or mobility-aware feedback. More recently, advanced frameworks like references [25,26] have emerged to counter false data injection and gradient-level privacy leakage by integrating blockchain with reputation-driven incentives. However, these schemes mainly protect the FL process itself and are not tightly coupled with source identity authentication, leaving room for forged-identity, Sybil, and cross-layer attacks [10].

### 2.3. Blockchain-Enabled Trust Management in IoV

Blockchain is widely used to support decentralized trust, traceability, and tamper resistance in IoV [6]. Closely related privacy-preserving data-sharing studies have also shown that protecting sensory information in IoV requires not only encryption but also careful control of identity exposure and data access paths [27]. To overcome the latency bottlenecks of conventional chain-based ledgers [28,29], DAG-based structures have emerged as a suitable alternative for asynchronous confirmation. Prior studies such as [8,11] show that DAG structures can improve scalability and remove the single-point bottleneck of centralized coordination, while the Tangle design remains a representative lightweight option for IoT and IoV environments [30]. Even so, most existing schemes store only communication data or FL data, rather than jointly supporting cross-layer evidence anchoring and fusion-trust evaluation.

### 2.4. Core Differences from Existing Related Work

Existing studies largely remain split between communication-layer authentication and application-layer FL security. Compared with TABMA [7], BATS [17], BEBA [18], DAG-FL [8], EBCFL [23], and [24], DAG-CTFL combines cross-layer identity authentication, cryptographic process reuse for secure transmission, and fusion-trust evaluation across the communication and learning layers. This design improves resistance to compound attacks while avoiding the extra cost of maintaining separate security mechanisms for the two layers. Furthermore, even when compared to the latest 2025–2026 blockchain-FL studies (e.g., TrustBlockFL [25] and FLARE [26]), which still rely on independent security stacks, DAG-CTFL uniquely achieves cryptographic process reuse to deeply couple communication security with federated learning protection. Table 1 makes this distinction explicit: most existing schemes focus on only one or two aspects, whereas DAG-CTFL jointly supports identity authentication, trust-aware access control, model-update reliability assessment, and cross-layer DAG-based evidence storage within a unified workflow.

## 3. System Model and Threat Model

### 3.1. System Model

**Figure 1:**
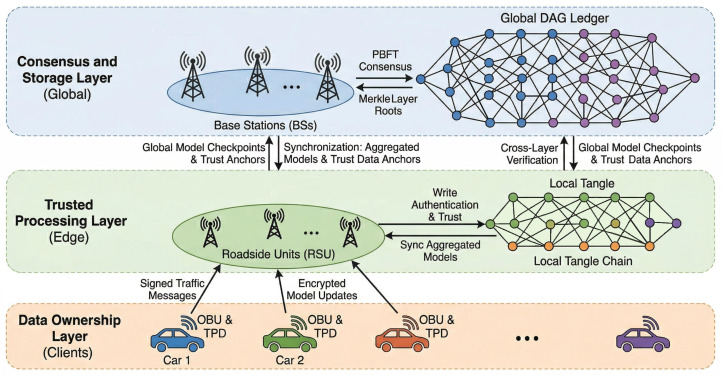
System model of the DAG-CTFL framework.

As shown in Figure 1, DAG-CTFL combines a three-tier entity architecture with a two-tier DAG ledger. This design separates data ownership, edge-side trust processing, and global consensus. It places latency-sensitive tasks such as identity authentication, trust evaluation, and local aggregation at the roadside edge, while using the two-tier ledger to support tamper-resistant storage and cross-layer synchronization of trust records and model data.

System Workflow Overview: To further clarify the data flow illustrated in Figure 1, the end-to-end operation of DAG-CTFL can be summarized as follows. The process starts at the Data Ownership Layer with local data collection, message signing, and model-update encryption at vehicles. It then proceeds to the Trusted Processing Layer for identity authentication, fusion-trust evaluation, and trust-driven local aggregation at RSUs, with records anchored to the Local Tangle. Finally, it reaches the Consensus and Storage Layer for cross-RSU synchronization, trust-aware PBFT consensus, and global-model dissemination among base stations to update the Global DAG Ledger. In this way, authentication, trust management, federated learning, and blockchain anchoring are organized into a closed-loop cross-layer workflow.

#### 3.1.1. Three-Tier Entity Architecture

DAG-CTFL organizes the system into three layers.

(1) Data Ownership Layer: Vehicles act as FL clients and message initiators. Equipped with On-Board Units (OBUs) and Tamper-Proof Devices (TPDs), they perform local data collection, model training, message signing, and encrypted transmission. Raw sensing data remain local, while only signed traffic messages and encrypted model updates are transmitted.
(2) Trusted Processing Layer: RSUs serve as trusted edge nodes and handle identity authentication, message verification, model-update decryption, fusion-trust evaluation, trust-based node filtering, local weighted aggregation, and local-ledger maintenance. They also synchronize aggregated models and trust records to the upper layer.
(3) Consensus and Storage Layer: Base stations (BSs) form a consortium blockchain cluster that maintains the global DAG ledger. This layer performs cross-RSU global aggregation and final confirmation through PBFT, supports cross-regional trust synchronization, and stores confirmed global model checkpoints together with key trust data for auditing.

#### 3.1.2. Two-Tier DAG Ledger Design

The two-tier DAG ledger separates local processing from global consensus to support cross-layer trust fusion.

(1) Local Tangle Chain: Maintained by the RSU cluster within a geographic region, the local ledger uses a DAG-based Tangle structure for high-concurrency and low-latency transaction processing. It stores authentication records, message-verification results, model-update metadata, and fusion trust values, and uses confidence-based tip selection for fast asynchronous confirmation.
(2) Global DAG Ledger: Maintained by base-station nodes across the network, the global ledger stores only PBFT-verified core data, including global-model checkpoints, cross-regional trust anchors, and Merkle roots of local ledgers. This hybrid DAG+PBFT design provides strong global consistency and Byzantine fault tolerance without sacrificing storage efficiency.

### 3.2. Threat Model

We consider five representative threats in IoV federated learning: external attacks, internal malicious-node attacks, honest-but-curious infrastructure attacks, cross-layer compound attacks, and long-term quantum threats. The assumptions are summarized as follows:

(1) External Attacks: Adversaries eavesdrop on open wireless channels and attempt forgery, tampering, replay, or impersonation attacks on traffic messages and model-update transmissions, without breaking the underlying cryptographic primitives.
(2) Internal Malicious Node Attacks: Legitimate but compromised vehicles may send false traffic messages, upload poisoned model updates, perform on-off evasion, or collude with other malicious nodes to manipulate trust evaluation and degrade global-model performance.
(3) Honest-but-Curious Infrastructure Attacks: RSU and base-station nodes follow the protocol but may try to infer sensitive information, such as local raw data or identity privacy, from received authentication records and model updates.
(4) Cross-Layer Compound Attacks: Attackers may first obtain network access through forged or abused identities and then launch application-layer poisoning or trust-manipulation attacks, thereby exploiting the gap between communication security and FL security.
(5) Quantum Adversary Threats: Although immediate large-scale quantum computers are not fully mature today, highly sensitive IoV infrastructure requires long-term forward secrecy to defend against the ‘harvest now, decrypt later’ threat strategy. Therefore, our hybrid post-quantum mechanism serves as a crucial transitional security design.

### 3.3. Security Requirements

Based on this threat model, DAG-CTFL should satisfy the following requirements:

(1) Authenticity and Integrity: The framework should verify the legitimacy of message and model-update sources and ensure transmission integrity.
(2) Freshness: The framework should identify and reject replayed or expired messages and model updates.
(3) Malicious-Node Resistance: The framework should detect and isolate persistent attackers, on–off attackers, colluding nodes, and poisoning participants.
(4) Privacy Preservation: The framework should protect raw-data privacy and identity privacy against inference by honest-but-curious infrastructure nodes.
(5) Availability and Robustness: The framework should tolerate a certain proportion of Byzantine nodes while maintaining system availability, trust reliability, and model robustness.
(6) Long-Term Security with Low Overhead: The framework should preserve the lightweight advantage required by vehicles and RSUs while providing incremental protection for future quantum threats.

## 4. Design of DAG-CTFL Framework

This section presents the core design of the DAG-CTFL framework, including six parts: overall workflow, core cryptographic fundamentals, lightweight collaborative authentication protocol, hybrid post-quantum enhancement, cross-layer trust management with DAG-based consensus coordination, and trust-based federated learning aggregation. For readability, the main mathematical symbols used in the protocol design and analysis are summarized in Table 2.

### 4.1. Overall Workflow of the Framework

The end-to-end workflow of DAG-CTFL connects identity authentication, trust evaluation, model training, and aggregation across the communication and learning layers. It is organized into six stages that operate sequentially and feed the updated trust results into the next round:

(1) System Initialization Stage: The Trusted Authority (TA) completes the generation of system public parameters, node registration, initial trust value allocation, and initial deployment of the two-tier DAG ledger.
(2) Trust Pre-Filtering Stage: After receiving the access request and FL participation request from a vehicle node, the RSU queries the node’s historical trust value from the local Tangle chain and performs pre-filtering. Only nodes whose trust values exceed the threshold are allowed to join the current communication and training round.
(3) Cross-Layer Collaborative Authentication Stage: Through the lightweight collaborative authentication protocol, the identity authentication of the vehicle node, message verification, and encrypted transmission of model updates are completed simultaneously, reusing the cryptographic operation process to reduce redundant overhead. For critical model-update and global-model dissemination messages, an additional hybrid post-quantum protection tag is attached to provide long-term security reinforcement.
(4) Cross-Layer Trust Update Stage: Based on authentication outcomes, message-authenticity feedback, and model-update quality, the RSU recalculates the node’s trust value and records both the updated score and the related metadata on the local Tangle chain.
(5) Trust-Based Federated Learning Aggregation Stage: Using the current trust values of participating nodes, the RSU assigns different aggregation weights to legitimate model updates and produces a local aggregated model, thereby reducing the influence of malicious updates on the global model.
(6) Global Consensus and On-Chain Stage: The base station cluster collects the local aggregated models of each RSU, completes global model aggregation and PBFT consensus, records the global model checkpoints and cross-regional trust anchor data to the global DAG ledger, and completes cross-chain data synchronization.

### 4.2. Core Cryptographic Fundamentals

This paper adopts the lightweight certificateless elliptic curve cryptography (CL-ECC) system as the underlying cryptographic foundation, which avoids the high computing overhead brought by bilinear pairing, and simultaneously solves the problems of certificate management and key escrow. To address the long-term quantum-security limitation of pure ECC, the framework further adopts a hybrid design principle: routine V2X messages still use CL-ECC authentication, while critical model-update and global-model dissemination messages are additionally protected by a lightweight post-quantum authentication component. The core cryptographic definitions are as follows:

Let *p* be a large prime number, and define an elliptic curve over the finite field $F_{p}$:

(1) $$E:y^{2}=x^{3}+ax+b\;\mathrm{mod}\;p,$$

where $a,b\in F_{p}$ and satisfy $4a^{3}+27b^{2}\neq 0\;\mathrm{mod}\;p$. Let *P* be a base point on the elliptic curve *E* with a large prime order *q*, which forms a cyclic additive group G. The Elliptic Curve Discrete Logarithm Problem (ECDLP) is computationally infeasible in the group G.

We define five collision-resistant secure hash functions: $h_{1},h_{2},h_{3},h_{4},h_{5}:{\left\{0,1\right\}}^{*}\to Z_{q}^{*}$, which are used for identity hashing, signature generation, integrity verification, and session key derivation, respectively. The TA generates the system master private key $s\in Z_{q}^{*}$ and the system master public key $P_{pub}=sP$, and publishes the system public parameters $\left\{p,a,b,P,q,P_{pub},h_{1},h_{2},h_{3},h_{4},h_{5}\right\}$.

For the hybrid enhancement, let PQ.KeyGen, PQ.Sign, and PQ.Verify denote the key generation, signing, and verification algorithms of a lightweight post-quantum signature primitive. The PQ primitive is not used for all routine broadcast messages, but only for critical messages that affect long-term FL integrity, thereby minimizing additional overhead while preserving a clear migration path toward post-quantum deployment.

### 4.3. Hybrid Post-Quantum Security Enhancement

To minimize changes to the original framework while addressing the post-quantum security shortcoming of pure ECC, DAG-CTFL adopts a hybrid enhancement strategy for critical messages rather than replacing the entire CL-ECC protocol stack. Specifically, ordinary V2X safety messages still follow the original certificateless ECC authentication flow, whereas model-update uploads, cross-RSU trust synchronization records, and global-model dissemination messages additionally carry a post-quantum authentication field.

For a critical message $M_{i}^{crit}$, the sender first generates the original collaborative authentication tuple based on CL-ECC, and then computes a post-quantum signature:

(2) $$\sigma_{i}^{pq}=\mathrm{PQ}.\mathrm{Sign}(sk_{i}^{pq},H(M_{i}^{crit}∥PID_{i}∥t_{i})),$$

where $sk_{i}^{pq}$ is the node’s post-quantum secret key. The transmitted message is extended as:

(3) $$M_{i}^{hyb}=\langle M_{i}^{crit},\sigma_{i}^{ecc},\sigma_{i}^{pq},t_{i},PID_{i}\rangle ,$$

At the receiver side, identity legitimacy and low-latency authentication are still first verified through the original CL-ECC procedure. Only when the message belongs to the critical-message set $M_{crit}$ does the receiver further execute $\mathrm{PQ}.\mathrm{Verify}(pk_{i}^{pq},\sigma_{i}^{pq},H(M_{i}^{crit}∥PID_{i}∥t_{i}))$. This staged verification strategy ensures that the framework retains lightweight operation in routine communication while providing stronger long-term protection for the FL process under future quantum adversaries.

Specifically, this hybrid mechanism is implemented via signature concatenation, which mathematically combines a mature classical algorithm (e.g., ECDSA) with a NIST-standardized post-quantum candidate (e.g., CRYSTALS-Dilithium). This dual-layer encryption ensures that the authentication remains secure even if one of the underlying mathematical hardness assumptions is compromised.

### 4.4. Lightweight Collaborative Authentication Protocol Design

The collaborative authentication protocol is the core of the framework, which realizes the process reuse of IoV secure message authentication and federated learning model update transmission. It is divided into three core stages: system initialization, node registration, and collaborative authentication and transmission.

#### 4.4.1. Stage 1: System Initialization

The TA executes the system initialization process, generates the elliptic curve parameters, system master public-private key pair, and hash functions defined above, and publishes the system public parameters. Meanwhile, it completes the registration of RSU and base station nodes, allocates public-private key pairs and initial permissions for each node, deploys the initial configuration of the local Tangle chain and global DAG ledger, and sets the initial trust value $T_{init}=0.6$ for all registered vehicle nodes.

#### 4.4.2. Stage 2: Vehicle Node Registration

The vehicle node $V_{i}$ sends a registration request to the TA, submitting its real identity $ID_{i}$ and user identity $UID_{i}$. After verifying the legitimacy of the identity, the TA performs the following operations:

1. Generate the login authentication information of the vehicle node:
  (4) $$A_{i}=h_{1}\left(UID_{i},ID_{i},s\right),\;B_{i}=h_{2}\left(PW_{i}\right)⊕A_{i},$$
  where $PW_{i}$ is the access password set by the node, which is stored in the on-board TPD.
2. Select a random number $k_{i}\in Z_{q}^{*}$, calculate $K_{i}=k_{i}P$, generate the pseudonym identity of the node $PID_{i}=ID_{i}⊕h_{3}\left(k_{i}P_{pub}\right)$, and the partial private key of the node $x_{i}=k_{i}+s$.
3. Transmit $\left\{A_{i},B_{i},K_{i},PID_{i},x_{i}\right\}$ to the TPD of the vehicle node through a secure channel for storage. The pseudonym $PID_{i}$ is also used as the unique address of the node in the blockchain to realize identity privacy preservation.

#### 4.4.3. Stage 3: Collaborative Authentication and Transmission Process

This process simultaneously completes vehicle-node authentication, traffic-message verification, and encrypted transmission of federated learning model updates, without running two separate protocol stacks. The specific steps are as follows:

Step 1: Collaborative Signing and Encryption of Messages and Model Updates. The vehicle node $V_{i}$ completes the collection of the local traffic message $M_{i}$ and the local federated learning model training, and obtains the model update $\Delta w_{i}=\theta_{local}-w_{g}$, where $w_{g}$ is the current global model parameter. The node performs the following operations:

1. Select a random number $c_{i}\in Z_{q}^{*}$, and calculate the temporary public key $C_{i}=c_{i}P$.
2. Generate a signature for the traffic message: calculate the hash value $h_{i}=h_{4}(PID_{i}∥M_{i}∥K_{i}∥C_{i}∥t_{v})$, where $t_{v}$ is the timestamp, the signature private key $\alpha_{i}=x_{i}+h_{i}c_{i}$, and the final signature $\sigma_{i}=\left\{C_{i},\alpha_{i}\right\}$.
3. Privacy preservation for model updates: add calibrated Gaussian noise to the model update to satisfy (ϵ,δ)-differential privacy, obtaining:
  (5) $$\Delta w_{i}^{\prime }=\Delta w_{i}+N\left(0,\sigma^{2}I\right).$$
  From an information-flow viewpoint, this perturbation increases the uncertainty of the released update and suppresses the mutual information that an honest-but-curious observer can extract about the original local training data.
4. Collaborative encryption: use the session key $K_{AES}=h_{5}(x_{i}∥t_{v}∥C_{i})$ derived from the same signature system to encrypt the noise-added model update through the AES-256 algorithm, obtaining the ciphertext $C_{model}=Enc_{AES-256}\left(\Delta w_{i}^{\prime },K_{AES}\right)$.
5. Send the data packet $\left\{M_{i},PID_{i},\sigma_{i},K_{i},t_{v},C_{model}\right\}$ to the corresponding RSU.

Step 2: Collaborative Verification and Decryption at the RSU. After receiving the data packet, the RSU executes the collaborative verification and decryption process, and simultaneously completes identity authentication, message verification, and model update decryption. The steps are as follows:

1. Freshness verification: check the validity of the timestamp $t_{v}$. If $|t_{r}-t_{v}|>\Delta T$ (where $t_{r}$ is the receiving time and ΔT is the freshness threshold), directly discard the data packet.
2. Trust pre-verification: query the current trust value $T_{i}$ corresponding to the node $PID_{i}$ from the local Tangle chain. If $T_{i}<T_{min}$ (trust threshold), directly discard the data packet without performing subsequent verification operations to reduce computing overhead.
3. Signature verification: calculate the hash value $h_{i}^{*}=h_{4}(PID_{i}∥M_{i}∥K_{i}∥C_{i}∥t_{v})$, and verify the equation:
  (6) $$\alpha_{i}P=K_{i}+P_{pub}+h_{i}^{*}C_{i},$$
  If the equation does not hold, it is determined as a forged message, the data packet is discarded, and the node’s trust value is updated.
4. Collaborative decryption: after the signature verification is passed, use the homologous session key $K_{AES}=h_{5}(x_{i}∥t_{v}∥C_{i})$ to decrypt the ciphertext $C_{model}$, obtain the model update $\Delta w_{i}^{\prime }$, and complete the trusted acquisition of the model update.

Step 3: Batch Collaborative Verification with Trust-Based Prioritization. For high-concurrency message and model-update transmission in IoV, the framework uses a batch-verification mechanism that prioritizes packets from more reliable nodes. The procedure is as follows:

1. After receiving *N* data packets, the RSU first filters the data packets of low-trust nodes through trust pre-verification, obtaining $N_{r}$ valid data packets.
2. Calculate the average trust value of the nodes corresponding to the valid data packets:
  (7) $$T_{avg}=\frac{\sum_{j=1}^{N_{r}}T_{j}}{N_{r}},$$
  Divide the data packets into a high-trust batch ($T_{j}\geq T_{avg}$) and a low-trust batch ($T_{j}<T_{avg}$), and sort the packets in descending order of trust value within each batch.
3. Perform batch verification on the two batches respectively. Taking the high-trust batch as an example, verify the equation:
  (8) $$\sum_{i=1}^{L}\alpha_{i}P=\sum_{i=1}^{L}K_{i}+L\cdot P_{pub}+\sum_{i=1}^{L}h_{i}^{*}C_{i},$$
  where *L* is the number of data packets in the batch. If the equation holds, all data packets in the batch are legitimate.
4. If the batch verification fails, use the binary search method to quickly locate invalid data packets and complete single-packet re-verification, avoiding the entire batch being discarded due to a single invalid message.

### 4.5. Cross-Layer Trust Management Mechanism

The cross-layer trust management mechanism is the main component used to detect and isolate internal attackers. It combines authentication behavior at the communication layer with model-contribution quality at the learning layer. Each node’s trust value is computed from direct and indirect trust, and a two-stage filtering strategy is used to match the timing of the federated learning process.

(1) Direct Trust Calculation

Direct trust is calculated from the interaction history between the node and the RSU, covering both communication-layer authentication behavior and learning-layer model contribution. The Gompertz function is used to capture the desired trust evolution pattern: gradual growth under normal behavior and rapid decline after malicious actions. In this sense, the Gompertz mapping acts as an uncertainty-to-trust transformation: uncertain or fluctuating cross-layer observations are converted into a conservative score, whereas sustained benign evidence reduces behavioral uncertainty and pushes the trust value upward in a measurable way.

The direct trust value $DT_{s}\left(i\right)$ of node $V_{i}$ in the *s*-th round is calculated as follows:

(9) $$DT_{s}\left(i\right)=e^{-\lambda \cdot e^{-\mu \cdot F_{s}\left(i\right)}},$$

where λ,μ are the shape parameters of the Gompertz function, with λ>1 and μ>0, and they control the rate of trust growth and decline; $F_{s}\left(i\right)$ is the node’s cross-layer behavior score, combining observations from the communication and learning layers, and is calculated as:

(10) $$F_{s}\left(i\right)=\eta \cdot \frac{N_{suc}^{com}}{N_{total}^{com}+\omega \cdot N_{fail}^{com}}+\left(1-\eta \right)\cdot \frac{N_{suc}^{fl}}{N_{total}^{fl}+\omega \cdot N_{fail}^{fl}},$$

where η∈[0,1] is the dimension-weight factor between the communication layer and the learning layer; $N_{suc}^{com}$ and $N_{fail}^{com}$ denote the numbers of successful and failed historical interactions at the communication layer, respectively; $N_{suc}^{fl}$ and $N_{fail}^{fl}$ denote the numbers of valid and malicious historical behaviors at the learning layer, respectively; and ω>1 is a penalty factor used to amplify the impact of malicious behaviors and accelerate trust reduction. Specifically, in the term ω·N, ω serves as a normalization scaling factor (defined as inversely proportional to the maximum local capacity). Therefore, ω·N explicitly represents the normalized interaction frequency of node *i*. This normalization is crucial to prevent highly active vehicles from disproportionately dominating the trust evaluation purely based on message volume.

(2) Indirect Trust Calculation

Indirect trust is calculated based on the feedback from surrounding vehicle nodes, which is used to improve the resistance to collusion attacks. Only valid feedback from high-trust nodes is adopted to avoid malicious nodes forging false feedback.

The indirect trust value $IT_{s}\left(i\right)$ of node $V_{i}$ in the *s*-th round is calculated as follows:

(11) $$IT_{s}\left(i\right)=\frac{\sum_{k\in N}T_{s-1}\left(k\right)\cdot r_{k\to i}\cdot \omega_{k}}{\sum_{k\in N}T_{s-1}\left(k\right)},$$

where N denotes the set of surrounding feedback nodes satisfying $T_{s-1}\left(k\right)\geq T_{min}$, $T_{s-1}\left(k\right)$ is the previous-round trust value of node *k*, and $r_{k\to i}\in \left[0,1\right]$ is the feedback on node $V_{i}$. The feedback credibility weight is defined as

(12) $$\omega_{k}=\frac{N_{acc}\left(k\right)}{N_{acc}\left(k\right)+N_{err}\left(k\right)+\xi },$$

in which $N_{acc}\left(k\right)$ and $N_{err}\left(k\right)$ denote the numbers of accurate and false feedbacks of node *k*, respectively, and ξ is a smoothing factor.

(3) Trust Value Calculation and Update

To support node selection in the two-tier DAG ledger, the trust value of node $V_{i}$ should reflect communication reliability, learning contribution quality, and penalties for abnormal behavior. After obtaining the direct and indirect trust terms, we define the trust value $T_{s}\left(i\right)$ of node $V_{i}$ in the *s*-th round as:

(13) $$T_{s}\left(i\right)=\beta \cdot DT_{s}\left(i\right)+\left(1-\beta \right)\cdot IT_{s}\left(i\right)-\gamma \cdot P_{s}\left(i\right),$$

where β∈[0,1] is the weight factor, which can be adjusted according to the scenario: in urban scenarios with large traffic volume and many feedback nodes, β can be reduced to improve the resistance to collusion attacks; in remote scenarios with small traffic volume, β can be increased to rely more on direct interaction results. $P_{s}\left(i\right)$ denotes the abnormal penalty item induced by replay attempts, invalid signature submissions, abnormal gradient deviations, or repeated low-quality model contributions, and γ>0 controls the penalty intensity.

To avoid trust value stagnation and adapt to the dynamic changes of node behavior, a time decay mechanism is introduced to update the trust value, while resisting on-off attacks:

(14) $$T_{s}^{new}\left(i\right)=\theta \cdot T_{s}\left(i\right)+\left(1-\theta \right)\cdot T_{s-1}\left(i\right),$$

where θ∈(0,1] is the decay factor, which gives priority to the latest behavior evaluation results, while avoiding violent fluctuations of the trust value. When the node switches from honest behavior to malicious behavior, the trust value will drop rapidly; when the node resumes honest behavior, the penalty of historical malicious behavior will limit the rapid recovery of the trust value, effectively resisting on-off attacks.

The updated trust value $T_{s}^{new}\left(i\right)$ is then reused by the ledger and consensus modules. Specifically, it affects transaction-reference priority in the local Tangle chain, committee admission in the global DAG ledger, and leader selection among qualified infrastructure nodes. In this way, behavior evaluation, trust updating, and consensus participation remain connected across rounds.

(4) Two-Stage Trust Filtering Mechanism

For the federated learning training process, a two-stage trust filtering mechanism is designed to solve the timing dependency problem between model similarity calculation and node filtering:

Stage 1: Pre-Training Filtering Stage: Before the start of each round of federated learning training, the RSU performs pre-filtering based on the node’s historical trust value ($T_{s-1}\left(i\right)$ updated in the previous round). Only nodes with $T_{s-1}\left(i\right)\geq T_{threshold}$ can participate in this round of training and receive the global model parameters.

Stage 2: Post-Training Trust Update Stage: After this round of training is completed, the RSU recalculates the node’s trust value based on authentication outcomes, message-verification results, model-update similarity, and contribution quality. The updated score is then recorded on the local Tangle chain and used for pre-filtering in the next round.

### 4.6. Two-Tier DAG Blockchain Consensus and Storage Mechanism

The two-tier DAG blockchain provides tamper-resistant storage and cross-regional synchronization for trust records and model data. It consists of two parts: local Tangle-chain processing and global DAG-ledger consensus.

(1) Local Tangle Chain Design

The local Tangle chain is jointly maintained by the RSU cluster in the area, adopting the Tangle DAG structure optimized for high-concurrency and low-latency local transaction processing. Each transaction corresponds to a DAG node, and a new transaction needs to verify two previous unconfirmed transactions (tips) without block packaging, realizing asynchronous and concurrent transaction processing.

The transaction structure stored in the local Tangle chain is as follows:

(15) $$\begin{array}{cc} Tx_{local}= & \langle PID_{i},T_{s}^{new}\left(i\right),hash\left(M_{i}\right),\\ \; & \;hash\left(\Delta w_{i}^{\prime }\right),t_{s},sign_{RSU}\rangle , \end{array}$$

which includes the node pseudonym, updated trust value, message hash, model update hash, timestamp, and RSU signature, ensuring that all data is tamper-proof and traceable.

The local Tangle chain adopts a confidence accumulation-based tip selection mechanism. The confidence of a transaction increases with the number of subsequent transactions that verify it. When the confidence reaches the preset threshold, the transaction is finally confirmed without global consensus, realizing millisecond-level transaction confirmation to meet the low-latency requirements of IoV.

To make local confirmation more robust against malicious transactions, the framework further introduces a tip-selection policy that takes node trust into account. For each candidate tip $tx_{j}$, its reference priority is defined as:

(16) $$\begin{array}{cc} W_{tip}\left(tx_{j}\right)= & \alpha_{1}\cdot Conf\left(tx_{j}\right)+\\ \; & \alpha_{2}\cdot T_{s}^{new}\left(v_{j}\right)-\alpha_{3}\cdot Risk\left(tx_{j}\right), \end{array}$$

where $Conf(tx_{j})$ is the current confidence of transaction $tx_{j}$, $T_{s}^{new}\left(v_{j}\right)$ is the latest trust value of the node that issued $tx_{j}$, and $Risk(tx_{j})$ is the anomaly score of the transaction. New transactions preferentially verify tips with higher $W_{tip}$, which helps limit the spread of low-trust or suspicious transactions in the local DAG while preserving asynchronous concurrency.

(2) Global DAG Ledger and Cross-Chain Consensus

The global DAG ledger is jointly maintained by base station nodes across the network and uses a hybrid mechanism that combines DAG storage with PBFT, thereby providing strong consistency and reliable storage for the global state. DAG-CTFL further incorporates trust-based committee admission and leader scheduling into this upper-layer consensus process. Cross-chain consensus is divided into two stages:

#### 4.6.1. Stage 1: Local State Submission

Within each time window, the RSU calculates the Merkle root $root_{B}$ for the set of transactions that have been finally confirmed in the local Tangle chain, generates a local state submission data packet including the Merkle root, the hash value of the local aggregated model, the timestamp, and the RSU signature, and sends it to the base station cluster. Only RSUs satisfying $T_{s}^{new}\left(r\right)\geq \tau_{RSU}$ are allowed to submit high-priority consensus packets, while low-trust RSUs are downgraded to delayed synchronization mode.

#### 4.6.2. Stage 2: PBFT Global Consensus and On-Chain Storage

The base station cluster executes PBFT to reach agreement on the local state submitted by each RSU and on the global model aggregation result. This process tolerates up to $\left\lfloor \left(n_{BS}-1\right)/3\right\rfloor$ Byzantine base-station nodes. Before PBFT starts, the cluster first forms a consensus committee from sufficiently reliable nodes:

(17) $$C_{s}=\left\{b_{k}|T_{s}^{new}\left(b_{k}\right)\geq \tau_{BS}\right\},$$

and the temporary leader is selected according to

(18) $$\begin{array}{cc} Leader_{s}= & \arg\max_{b_{k}\in C_{s}}\\ \; & \left(\rho_{1}T_{s}^{new}\left(b_{k}\right)+\rho_{2}Q_{s}\left(b_{k}\right)-\rho_{3}D_{s}\left(b_{k}\right)\right), \end{array}$$

where $Q_{s}\left(b_{k}\right)$ denotes the historical consensus quality of node $b_{k}$ and $D_{s}\left(b_{k}\right)$ denotes the delay penalty. After the committee is formed, PBFT is executed to finalize the global state. Once consensus is reached, a global block is generated with the following structure:

(19) $$\begin{array}{cc} Block_{global}= & \langle hash\left(w_{g}^{new}\right),prev_{hash},\\ \; & \;H(root_{B}^{1}∥root_{B}^{2}∥\cdots ∥root_{B}^{F}),t_{s},sign_{PBFT}\rangle , \end{array}$$

which includes the hash of the new global model, the hash of the previous block, the Merkle root anchor of the local ledger of each RSU, the timestamp, and the PBFT consensus signature. The block is appended to the global DAG ledger to realize tamper-proof storage of the global state and cross-regional trust data synchronization.

To ensure the scalability of the global layer in large-scale urban scenarios, the communication complexity of the PBFT-based consensus must be addressed. While standard PBFT requires $O(N^{2})$ message exchanges among *N* nodes, DAG-CTFL mitigates this by restricting consensus participation to a dynamically selected committee $C_{s}$, as defined in Equation (17). By maintaining a constant or slowly-growing committee size $C=|C_{s}|$, where C≪N, the communication overhead is constrained to $O(C^{2})$. This hierarchical consensus structure allows the framework to decouple global state synchronization from the total number of BSs in the network, thereby preventing communication bottlenecks and ensuring high throughput even as the city-wide deployment scale expands.

To improve the timeliness of trust updates, the framework also introduces an enhanced PoW-based miner-election mechanism. The mining difficulty of an RSU is linked to the fluctuation range of trust values in its area: larger trust changes lead to lower mining difficulty and faster synchronization of key trust records. As a result, local DAG processing, upper-layer PBFT coordination, and trust updating operate as a coordinated mechanism rather than as isolated modules.

### 4.7. Trust-Based Federated Learning Aggregation Process

Based on the cross-layer trust value, the framework uses a trust-based federated learning aggregation algorithm instead of the uniform averaging strategy in traditional FedAvg. This design reduces the impact of malicious model updates and improves robustness against poisoning attacks.

The core process is shown in Algorithm 1, with the following specific steps:
**Algorithm 1** Trust-Based Federated Learning Aggregation Algorithm**Require:** Global model $w_{g}$, trust threshold $T_{min}$, training rounds *R***Ensure:** Final global model $w_{g}^{final}$ 1:  **for** r=1 to *R* **do** 2:  RSU retrieves historical trust values $T_{r-1}\left(i\right)$ from local Tangle; 3:  $V_{selected}\leftarrow \left\{v_{i}|T_{r-1}\left(i\right)\geq T_{min}\right\}$; 4:  Distribute $w_{g}$ to nodes in $V_{selected}$; 5:  **for** each node $v_{i}\in V_{selected}$ **do** 6:     Perform local training $\Delta w_{i}$; 7:     Add DP noise: $\Delta w_{i}^{\prime }=\Delta w_{i}+N\left(0,\sigma^{2}I\right)$; 8:     Encrypt and send $\Delta w_{i}^{\prime }$ to RSU; 9:  **end for**10:  RSU evaluates authentication results, message validity, and model-update quality;11:  Compute $F_{r}\left(i\right)$, $DT_{r}\left(i\right)$, $IT_{r}\left(i\right)$, penalty $P_{r}\left(i\right)$, and fused trust $T_{r}\left(i\right)$;12:  Update trust value $T_{r}^{new}\left(i\right)=\theta T_{r}\left(i\right)+\left(1-\theta \right)T_{r-1}\left(i\right)$;13:  Record updated trust evidence and $T_{r}^{new}\left(i\right)$ on the local Tangle ledger;14:  RSU filters valid set $V_{valid}=\left\{v_{i}|T_{r}^{new}\left(i\right)\geq T_{min}\right\}$;15:  **Local Aggregation:** $\Delta w_{RSU}=\frac{\sum_{v_{i}\in V_{valid}}T_{r}^{new}\left(i\right)\cdot \Delta w_{i}^{\prime }}{\sum_{v_{i}\in V_{valid}}T_{r}^{new}\left(i\right)}$;16:  BS collects results and performs Global Aggregation;17:  $w_{g}\leftarrow w_{g}+\Delta w_{global}$;18:  Record model checkpoints and trust anchors on Global DAG Ledger;19: **end for**20: **return** $w_{g}$

Step 1: Local Model Training. Vehicle nodes that pass the pre-filtering train the model based on the local dataset, obtain the model update $\Delta w_{i}^{\prime }$, and send it to the RSU after encryption.

Step 2: Trust Evaluation and Update. After receiving the encrypted model updates, the RSU evaluates authentication results, message validity, and model-update quality. It then computes $F_{s}\left(i\right)$, $DT_{s}\left(i\right)$, $IT_{s}\left(i\right)$, and $P_{s}\left(i\right)$ according to the trust model, obtains $T_{s}\left(i\right)$, updates it to $T_{s}^{new}\left(i\right)$ through the time-decay rule, and records the updated trust evidence on the local Tangle ledger.

Step 3: Valid Node Filtering. After decrypting the model update, the RSU filters the valid node set $V_{valid}$ with $T_{s}^{new}\left(i\right)\geq T_{min}$ based on the updated real-time trust value of the node.

Step 4: Trust-Based Local Aggregation. The RSU aggregates the model updates of valid nodes using trust-derived weights to obtain the local aggregated model update:

(20) $$\Delta w_{RSU}=\frac{\sum_{i\in V_{valid}}T_{s}^{new}\left(i\right)\cdot \Delta w_{i}^{\prime }}{\sum_{i\in V_{valid}}T_{s}^{new}\left(i\right)}.$$

Step 5: Global Model Aggregation. The base station cluster collects the local aggregated model updates of each RSU, performs a second global aggregation to obtain the global model update:

(21) $$\Delta w_{global}=\frac{1}{F}\sum_{j=1}^{F}\Delta w_{RSU_{j}},$$

where *F* is the number of RSUs participating in the aggregation. Finally, the global model is updated as $w_{g}^{new}=w_{g}+\Delta w_{global}$, completing this round of training.

## 5. Security Analysis

This section verifies the security of DAG-CTFL through formal verification, cryptographic proofs, and attack-resistance analysis, while clarifying the role of the hybrid post-quantum enhancement for critical messages. To provide a precise formalization of our security assumptions, we adopt the standard Dolev-Yao (DY) threat model. Under this model, an adversary exerts complete control over the public communication channels—capable of intercepting, modifying, injecting, and replaying V2X messages and federated learning model updates. However, the adversary is polynomially bounded and cannot break the underlying mathematical hardness of the cryptographic primitives without possessing the legitimate private keys.

### 5.1. Formal Security Verification

In this paper, ProVerif 2.05 is used to perform formal security verification on the collaborative authentication protocol of the framework, based on the Dolev-Yao adversary model. The adversary can monitor, intercept, tamper with, and replay all messages in the network, but cannot crack the elliptic curve discrete logarithm problem and the collision resistance of hash functions.

The verified attributes include identity authenticity, message integrity, replay resistance, and identity privacy. The ProVerif results are summarized in Table 3.

The results show that the proposed protocol satisfies the required security attributes and resists forgery, tampering, and replay. For critical-message flows, ECC-based authentication and the post-quantum tag jointly provide layered integrity protection.

### 5.2. Cryptographic Security Proofs

Under the Random Oracle Model, the proposed certificateless signature scheme is shown to satisfy EUF-CMA security against the two standard adversaries in certificateless cryptography: Type I adversaries, which can replace public keys but do not know the master secret, and Type II adversaries, which know the master secret but cannot replace public keys.

**Theorem** **1.** *Under the Random Oracle Model, if the Elliptic Curve Discrete Logarithm Problem (ECDLP) is computationally infeasible in the cyclic group G, the proposed signature scheme can resist adaptive chosen message attacks from Type I adversaries and satisfies EUF-CMA security.*

**Proof.**  Assume that the Type I adversary $A_{I}$ can forge a valid signature with a non-negligible probability ϵ. We can construct a simulator B that uses the forging capability of $A_{I}$ to solve the ECDLP problem. Given an ECDLP instance (P,Q=sP), the goal of the simulator B is to solve *s*.

The simulator B sets the system master public key $P_{pub}=Q$, simulates the system environment, and provides $A_{I}$ with hash oracle, partial private key query, public key query, and signature query oracle services. Finally, $A_{I}$ outputs a valid forged signature $\left(PID^{*},M^{*},\sigma^{*}=\left(C^{*},\alpha^{*}\right)\right)$ without making a signature query for this message. According to the signature verification equation:

(22) $$\alpha^{*}P=K^{*}+sP+h^{*}C^{*},$$

we can derive:

(23) $$s=\alpha^{*}-k^{*}-h^{*}c^{*}\;\mathrm{mod}\;q,$$

where $K^{*}=k^{*}P$ and $C^{*}=c^{*}P$. The simulator B successfully solves the solution *s* of the ECDLP instance.

Under the Random Oracle Model, the success probability of the simulator is $\epsilon^{\prime }\geq \left(1-\frac{q_{s}}{q_{h}}\right)\cdot \epsilon$, where $q_{s}$ is the number of signature queries and $q_{h}$ is the number of hash queries. If the forgery probability of $A_{I}$ is non-negligible, the probability of the simulator solving the ECDLP problem is also non-negligible, which contradicts the computational infeasibility of ECDLP. Therefore, the scheme can resist forgery attacks from Type I adversaries. □

**Theorem** **2.** *Under the Random Oracle Model, if the ECDLP problem is computationally infeasible in the cyclic group G, the proposed signature scheme can resist adaptive chosen message attacks from Type II adversaries and satisfies EUF-CMA security.*

**Proof.**  Consistent with the proof logic of Theorem 1, the Type II adversary $A_{II}$ knows the system master private key *s*, and the simulator B can use its forging capability to solve $k^{*}$ in the ECDLP instance, which contradicts the computational infeasibility of ECDLP. Therefore, the scheme can resist forgery attacks from Type II adversaries. □

### 5.3. Security Analysis of Hybrid Construction

To address the potential long-term threats posed by quantum adversaries and to ensure the robustness of the framework, DAG-CTFL incorporates a hybrid signature mechanism for critical messages $M_{crit}$ (e.g., global model dissemination and cross-regional trust anchors). The security of this hybrid construction is analyzed as follows.

(1) Formalization of the Hybrid Signature Model

For any critical message $M\in M_{crit}$, the hybrid signature is defined as a tuple $\Sigma_{hyb}=\langle \sigma_{ecc},\sigma_{pq}\rangle$, where $\sigma_{ecc}$ is the certificateless ECC-based signature generated in (4), and $\sigma_{pq}$ is the post-quantum signature generated in (2). The global verification function $V_{hyb}$ at the receiver side (RSUs or BSs) is defined as:

(24) $$V_{hyb}\left(M,\Sigma_{hyb}\right)=V_{ecc}\left(M,\sigma_{ecc}\right)\wedge V_{pq}\left(M,\sigma_{pq}\right),$$

where ∧ denotes the logical AND operation. A message is considered authentic if and only if both underlying signature schemes are verified successfully.

(2) Resistance to Downgrade Attacks

A downgrade attack occurs if an adversary can force the system to bypass the stronger security layer (PQ) and rely solely on the legacy layer (ECC). In DAG-CTFL, the protocol logic strictly defines the set $M_{crit}$. The verification logic at the BSs cluster and RSUs is hardcoded to trigger $V_{pq}$ for any message flagged as a model update or trust synchronization record.Since the verification is sequential and the PQ-tag is non-optional for $M_{crit}$, an adversary who successfully solves the Elliptic Curve Discrete Logarithm Problem (ECDLP) to forge $\sigma_{ecc}$ will still fail the total verification $V_{hyb}$ because the computationally infeasible post-quantum primitive PQ.Sign remains unbroken. Thus, the integrity of the cross-layer aggregation and global consensus is lower-bounded by the security strength of the PQ component:

(25) $$Adv_{hyb}^{EUF-CMA}\leq \min\left(Adv_{ecc}^{EUF-CMA},Adv_{pq}^{EUF-CMA}\right).$$

(3) Cross-Protocol Vulnerability Discussion

To avoid cross-protocol vulnerabilities where an adversary might use a signature from one layer to spoof another, DAG-CTFL employs domain separation. The input to the hash functions $h_{4}$ (for ECC) and *H* (for PQ) includes distinct protocol identifiers ($PID_{i}$) and unique timestamps ($t_{i}$). This ensures that even if the underlying messages are identical, the resulting signature bits are cryptographically decoupled. Furthermore, the session key $K_{AES}$ used for model update encryption is derived from the ECC state, but its integrity is anchored by the hybrid signature, preventing unauthorized access even in the event of partial key leakage.

### 5.4. Attack Resistance Analysis

The framework resists the main attacks considered in Section 3. Internal malicious nodes are quickly isolated through the two-dimensional trust mechanism, which combines communication behavior and model contribution quality; experiments show a detection rate above 93.2% with a false positive rate below 1.8%. The time-decay update further limits on-off evasion, while indirect trust weighted by feedback credibility suppresses collusion. Honest-but-curious servers are mitigated through differential privacy and encrypted transmission. Cross-layer compound attacks are constrained because forged identities fail trust pre-filtering and authenticated poisoning attempts trigger rapid trust reduction. At the infrastructure layer, the PBFT-based global DAG ledger tolerates up to $\lfloor \left(n_{BS}-1\right)/3floor$ Byzantine base stations, while the local Tangle chain avoids centralized single-point failure. For long-term integrity, critical FL-control messages carry post-quantum authentication evidence in addition to CL-ECC protection. Finally, trust-driven tip selection, committee admission, and leader election reduce the influence of low-trust nodes on both local and global consensus.

## 6. Experimental Results and Analysis

This section evaluates DAG-CTFL from four aspects: authentication latency and communication overhead, model robustness in the application-layer FL task, trust-detection capability, and batch-authentication message utilization. To better match the communication focus of the framework, the discussion emphasizes protocol reuse, batch verification, scalability, and the cost of cross-layer security protection. It should be noted that to strictly isolate the performance impact of the cross-layer cryptographic coupling and ensure exact reproducibility, the simulations in this study were conducted under highly controlled, fixed-seed settings. Consequently, the reported performance metrics represent deterministic baseline capabilities, effectively minimizing statistical variance across execution runs under these specific configurations.

### 6.1. Experimental Environment and Parameter Settings

Experiments are conducted on a workstation with an Intel Core i9-13900K CPU, an NVIDIA RTX 4090 GPU, and 32 GB memory, using Python 3.9, PyTorch 2.0, and ProVerif 2.05. The IoV federated learning scenario includes 20 vehicle clients, 4 RSUs, and 3 base stations over 50 communication rounds, with 10 clients selected per round. Local training uses SGD with learning rate 0.01, batch size 64, and 3 local epochs. The main settings are summarized below.

(1) Datasets and model settings: Two benchmark datasets represent IoV learning tasks with different complexity.

MNIST handwritten digit dataset: A multi-layer perceptron (MLP) with two hidden layers of 128 and 64 neurons is used. This setting represents lightweight in-vehicle tasks.

CIFAR-10 image classification dataset: A convolutional neural network (CNN) with two convolutional layers and two fully connected layers is employed, together with batch normalization and Dropout. This setting emulates more complex perception tasks at RSUs or edge-assisted vehicular nodes.

To capture the on-independent and identically distributed (Non-IID) property of IoV data, we partition the datasets across 20 clients using a Dirichlet distribution with concentration parameter α=0.5. This yields heterogeneous label distributions that better match realistic vehicular data collection.

(2) Attack scenario settings: We evaluate four malicious-client ratios: 5%, 10%, 15%, and 20%. Malicious clients inject forged messages at the communication layer and perform Gaussian-noise injection, gradient manipulation, and zero-value updates at the learning layer. Specifically, these learning-layer attacks include targeted label flipping, zero-value updates, and severe gradient manipulation via Gaussian noise injection with a high variance ($\sigma_{malicious}^{2}=5.0$). This high-variance noise is intentionally designed to rigorously test the limits of the aggregation algorithms; notably, when combined with the inherent differential privacy noise added by schemes like DP-FedAvg, it causes the signal-to-noise ratio to drop below the convergence threshold, explaining the cliff-like accuracy collapse observed at the 20% malicious ratio. We also simulate on-off attacks and 3-node collusion.

Privacy-Utility Trade-off Analysis: Regarding the differential privacy mechanism, there is an inherent trade-off between privacy protection and model utility. A smaller privacy budget ϵ injects larger DP noise, offering stronger theoretical privacy against gradient inference attacks, but inevitably degrades the signal-to-noise ratio and model convergence. DAG-CTFL mitigates this rigid trade-off by dynamically coupling the privacy budget with the cross-layer trust evaluation. By isolating untrustworthy updates before aggregation, the framework avoids the need to apply excessively small ϵ values across the entire network, successfully maintaining high global utility (e.g., 86.72% accuracy) while ensuring robust privacy protection.

(3) Comparison scheme settings: The baselines are organized in two dimensions.

Authentication performance comparison: EIDA [15], CASA [16], RSMA [31], and TABMA [7] are used as quantitative baselines for cryptographic overhead, batch verification efficiency, and message utilization. BATS [17] and the protocol in [18] are used only as qualitative references because their reported settings differ.

Application-layer robustness comparison: FedAvg [32], DSSFL [33], DP-FedAvg [34], and DAG-FL [8] are used as quantitative baselines for convergence, accuracy, and poisoning robustness. EBCFL [23] and the mobility-aware reputation-based scheme in [24] are used as qualitative references when a strictly unified simulation environment is unavailable.

Accordingly, the latency, utilization, and accuracy tables include only quantitatively reproducible baselines under the same platform, whereas Table 1 supports qualitative architectural comparison. In addition, the reported results are later interpreted from a component-oriented perspective to clarify the roles of trust fusion, the two-tier DAG architecture, and hybrid post-quantum enhancement.

(4) Core parameter settings: The main parameters are: trust threshold $T_{min}=0.5$; Gompertz-function parameters λ=2.2 and μ=1.3; penalty factor ω=3; trust weight β=0.7; trust-penalty coefficient γ=0.15; local tip-selection coefficients $\left(\alpha_{1},\alpha_{2},\alpha_{3}\right)=\left(0.45,0.40,0.15\right)$; global leader-selection coefficients $\left(\rho_{1},\rho_{2},\rho_{3}\right)=\left(0.50,0.30,0.20\right)$; differential privacy noise scale σ=0.05; privacy budget ϵ≈1.18; and freshness threshold ΔT=120 s. Only critical control messages carry an additional PQ authentication field, which improves long-term integrity without adding unnecessary cost to ordinary messages.

### 6.2. Communication and Computing Overhead Performance Analysis

(1) Single-Message Verification Overhead

Table 4 compares the core cryptographic operations for single-message signing and verification. DAG-CTFL uses 1 scalar multiplication for signing and 2 scalar multiplications plus 2 point additions for verification, which is the same operation pattern as TABMA and RSMA. Under the unified implementation setting, its measured single-message verification latency is 0.438 ms. This is more than 97% lower than pairing-based schemes, 8.2–56.1% lower than the ECC-based baselines, and 3.1% lower than TABMA. Therefore, the proposed design keeps the per-message authentication cost low enough for dense vehicular communication.

(2) Batch Verification Overhead

As the batch size increases, DAG-CTFL consistently keeps the lowest batch-verification latency among the evaluated schemes. At a batch size of 500, its latency is 192.0±3.1 ms, with a 95% confidence interval of [191.8, 192.2] ms over 1000 trials. This value is 19.2%, 56.4%, 14.1%, and 4.2% lower than EIDA, CASA, RSMA, and TABMA, respectively. More importantly, even the upper bound of the confidence interval remains well below the 300 ms security-message latency threshold defined by 5GAA. This indicates that the proposed framework can still support high-concurrency IoV authentication when message arrivals become dense.

We also evaluate the more demanding re-verification stage when invalid messages are mixed into a batch. When the batch size is 200 and the malicious-message ratio is 10%, DAG-CTFL records 234.2 ms in the worst case and 101.8 ms in the best case. The worst-case latency is 57.1% lower than that of the best comparison scheme, and the best-case latency is 8.3% lower than TABMA. This reduction comes from the trust-aware batch partition mechanism: low-trust messages are filtered early, so the probability of full-batch failure decreases and the subsequent re-verification cost is reduced.

**Figure 2:**
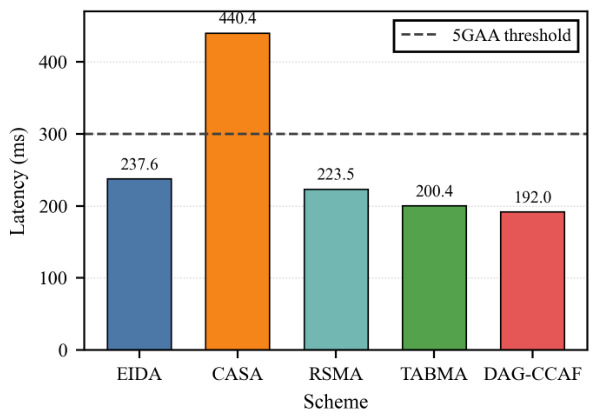
Batch verification latency at batch size 500.

Figure 2 visualizes the latency results at the largest evaluated batch size, namely 500 messages. The figure is used to illustrate the numerical comparison discussed above, rather than to introduce a different experiment. It shows that DAG-CTFL has the lowest latency among all schemes and remains below the 5GAA 300 ms threshold, whereas CASA has the highest latency. Together with Table 4, this figure shows that the reduced cryptographic cost of DAG-CTFL is reflected not only at the single-message level but also in end-to-end batch verification.

(3) Communication Overhead

The single-message payload of DAG-CTFL is 128 bytes, which meets the ETSI TS 103 097 payload limit and stays consistent with mainstream IoV authentication schemes. In the FL scenario, the proposed cross-layer protocol reuses key negotiation, signature, and verification procedures for both V2X messages and model-update transmission. As a result, it avoids the duplicated signaling cost of two independent protocol stacks. Quantitatively, DAG-CTFL reduces the per-round communication overhead by about 6.5% compared with an independent dual-protocol design, introduces only a 5.8% increase relative to standard FedAvg, and reduces communication overhead by 2.1% compared with DAG-FL [8]. These results indicate that the framework keeps communication cost under control while adding cross-layer security.

The hybrid post-quantum enhancement is activated only for the critical-message set $M_{crit}$ rather than for all V2X packets. Hence, the extra communication and verification overhead is bounded and mainly appears in model-update and global-model synchronization phases, where RSUs and base stations have stronger computing capability. This selective activation preserves the lightweight nature of the framework while improving long-term integrity protection.

Theoretical Complexity Analysis: To complement the empirical measurements, the asymptotic complexity of DAG-CTFL is summarized as follows. (1) Computational Complexity: For *N* vehicles in a region, the RSU’s batch verification requires O(N) elliptic curve point additions, effectively avoiding O(N) computationally expensive bilinear pairing operations. Client-side signing remains strictly O(1). (2) Communication Complexity: At the global consensus layer, by restricting PBFT participation to a trusted committee of size *C*, the communication overhead is strictly bounded to $O(C^{2})$, effectively avoiding the $O(N_{BS}^{2})$ bottleneck common in large-scale deployments. (3) Storage Complexity: Benefiting from the two-tier DAG architecture, end vehicles only need to store O(1) key states. Meanwhile, RSUs and BSs utilize periodic PBFT checkpoints and Merkle root anchoring to prune historical Tangle transactions, ensuring that the active storage overhead per infrastructure node remains manageable and scalable at O(1) relative to the continuously growing time steps.

### 6.3. Model Performance and Robustness Analysis

(1) Convergence Performance in Benign Scenarios

**Figure 3:**
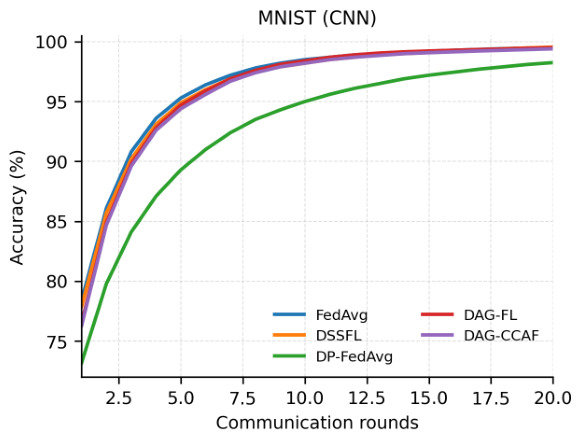
Model convergence curves on the MNIST dataset in the benign scenario.

**Figure 4:**
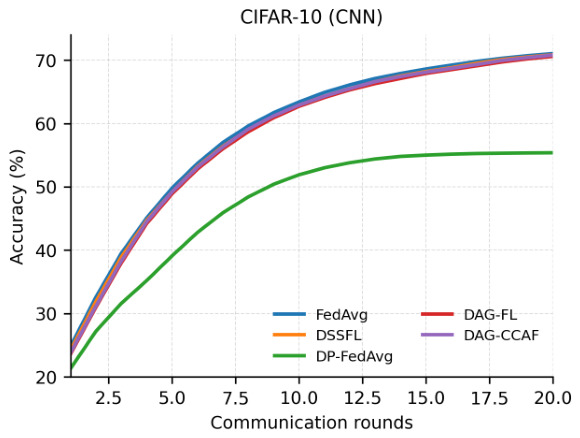
Model convergence curves on the CIFAR-10 dataset in the benign scenario.

Table 5 summarizes the final model accuracy in benign settings, while Figure 3 and Figure 4 show how the models converge over communication rounds on MNIST and CIFAR-10, respectively. Here FL serves as the application-layer workload used to test whether the proposed communication-security design introduces excessive utility loss. On MNIST, DAG-CTFL reaches 97.90% with MLP and 99.40% with CNN, remaining very close to FedAvg and DAG-FL and still outperforming DP-FedAvg. On CIFAR-10, it reaches 63.50% with MLP and 70.80% with CNN, which is 8.2–10.3 percentage points higher than DP-FedAvg. These results indicate that the proposed cross-layer authentication framework causes only limited utility loss in benign settings.

The two convergence figures provide a more detailed view of Table 5. Figure 3 shows that on MNIST, DAG-CTFL converges almost at the same rate as DAG-FL and remains close to FedAvg in the later rounds. Figure 4 shows a similar trend on the more difficult CIFAR-10 task: DAG-CTFL stays stable throughout training and consistently remains above DP-FedAvg. The 95% confidence intervals of DAG-CTFL are [97.59, 98.21]% for MNIST-MLP, [99.26, 99.54]% for MNIST-CNN, [62.66, 64.34]% for CIFAR-10-MLP, and [70.09, 71.51]% for CIFAR-10-CNN. Therefore, the added cross-layer protection does not destabilize the training process.

(2) Robustness in Malicious Scenarios

Table 6 first summarizes the MNIST-MLP accuracy under different malicious-node ratios. As the attack intensity increases from 5% to 20%, FedAvg, DSSFL, and DP-FedAvg degrade rapidly, whereas DAG-FL and DAG-CTFL remain much more stable. For example, at 15% malicious nodes, DAG-CTFL still reaches 86.72±0.64% on MNIST-MLP, with a 95% confidence interval of [85.92, 87.52]%. On the harder CIFAR-10 task, the framework also remains robust: at 15% malicious nodes, it still achieves 53.2±0.9% accuracy with MLP and 63.4±0.8% with CNN, while preserving stable late-stage convergence. This result is important mainly because it shows that the communication-oriented authentication design does not sacrifice application-layer robustness under adversarial settings.

**Figure 5:**
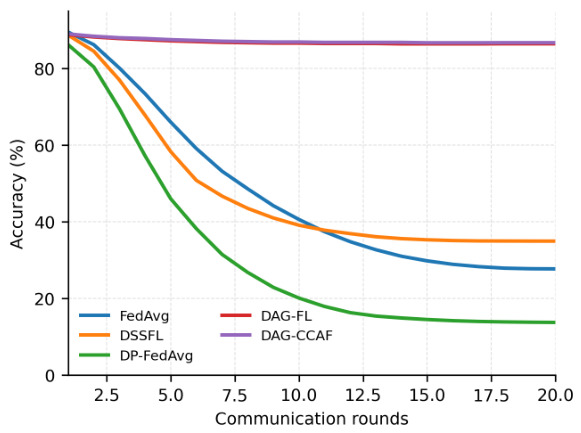
Model convergence curves under 15% malicious nodes.

Figure 5 corresponds to the 15% malicious-node setting and explains the dynamic behavior behind the numerical results. The three baseline schemes without strong trust-guided filtering suffer obvious accuracy collapse as training proceeds. By contrast, DAG-FL and DAG-CTFL keep a much smoother convergence trend, and DAG-CTFL ends with the highest final accuracy. This result shows that the proposed communication-learning joint defense helps suppress poisoned updates over time rather than only improving the final round result.

**Figure 6:**
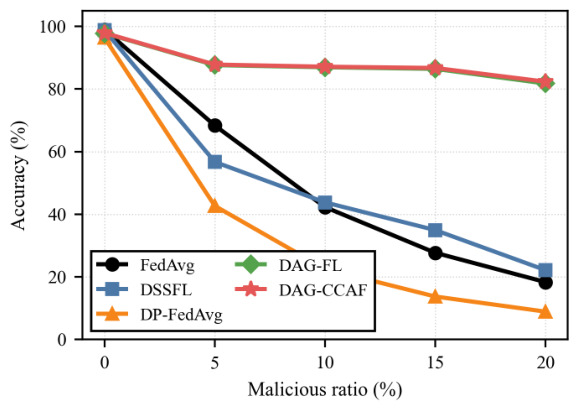
Final model accuracy versus malicious-node ratio.

Figure 6 summarizes the final accuracy as the malicious-node ratio increases. Its role is to show the overall robustness trend across attack intensities, complementing Figure 5, which focuses on one representative case. As the malicious-node ratio grows, the performance gap between DAG-CTFL and the weaker baselines widens, showing that the proposed framework degrades more gracefully under stronger attacks.

### 6.4. Trust Mechanism Detection Performance Analysis

The trust-management module is evaluated under 20% malicious nodes, including both on-off attacks and 3-node collusion. In this setting, the trust-management module of DAG-CTFL achieves 93.2% recall, 93.7% precision, and a 93.4% F1-score, while keeping the false positive rate below 1.8%. These results indicate that the module can identify most malicious participants without excessively penalizing benign nodes.

**Figure 7:**
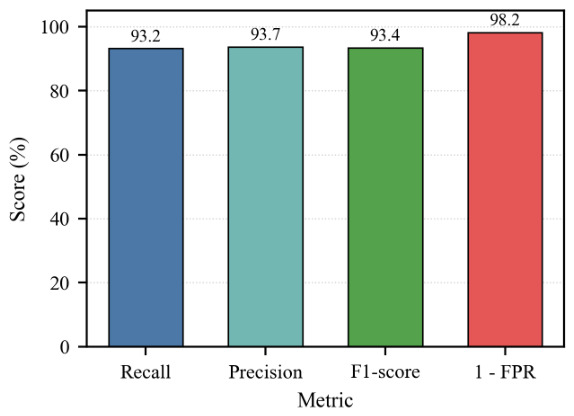
Detection performance of the trust-management module under malicious behaviors.

Figure 7 visualizes these detection metrics. It confirms that recall, precision, and F1-score all stay above 93%, whereas the false positive rate remains below 2%. Combined with the threshold-based trust decline observed in the attack simulations, this result supports the effectiveness of trust-driven participant scheduling.

Parameter Selection and Sensitivity: To validate the robustness of the trust configuration, we evaluated key parameters under different attack scenarios. Results show that varying the Gompertz shape parameter λ∈[2.0,2.5] and penalty coefficient γ∈[0.1,0.2] causes only a marginal fluctuation (±1.8%) in the F1-score. In practical deployments, these parameters serve as adaptive levers for different IoV scenarios. For instance, in dense urban intersections with high traffic volumes, administrators should configure higher γ and λ values to implement a stricter penalty mechanism for rapid malicious node isolation. Conversely, in sparse highway scenarios where interactions are brief and infrequent, increasing the direct trust weight β ensures that immediate local observations are prioritized. This demonstrates that DAG-CTFL is both structurally robust and highly adaptable to diverse traffic environments.

### 6.5. Batch Authentication Message Utilization Analysis

Table 7 shows the average message utilization under different malicious-node ratios. DAG-CTFL reaches 0.645, 0.565, and 0.526 at 10%, 20%, and 30%, respectively, and outperforms both EIDA and TABMA at all three attack intensities. This means that when invalid messages appear in a batch, the proposed framework can retain a larger proportion of valid messages for effective use.

This gain comes from two design choices. First, the trust-aware pre-filtering stage removes some low-trust malicious messages before batch verification starts. Second, the partition-and-binary-search re-verification procedure avoids discarding an entire batch because of one invalid message. At the 20% malicious-node ratio, the 95% confidence interval of DAG-CTFL utilization is [0.550, 0.580], which remains above the mean values of EIDA and TABMA. Therefore, even when the malicious-node ratio increases, the cross-layer authentication framework can still preserve a higher valid-message utilization rate.

## 7. Conclusions

This paper proposes DAG-CTFL, a DAG blockchain-enabled cross-layer authentication framework for trustworthy IoV federated learning. The framework is designed around communication efficiency as well as security: it reuses authentication operations across V2X messaging and model-update delivery, supports low-latency batch verification, and manages cross-layer trust evidence through a scalable two-tier DAG architecture. By integrating identity authentication, trust evaluation, and model-contribution assessment, DAG-CTFL reduces protocol redundancy and improves resistance to cross-layer attacks. Security analysis and experiments show that the proposed framework achieves a practical balance among authentication latency, communication overhead, robustness, and scalability. Specifically, it reduces batch verification latency by up to 56.4% and maintains 86.72% accuracy under 15% attacks. However, deploying DAG-CTFL in real IoV environments faces practical challenges. First, the framework must overcome the hardware computation constraints of legacy OBUs under high mobility. Second, while our current evaluations use standard benchmarks (MNIST/CIFAR-10) and fixed-seed simulations to strictly evaluate deterministic architectural baselines, real-world deployments inherently introduce significant statistical variance due to unpredictable channel fluctuations. Therefore, future work will optimize the framework for resource-constrained devices and establish a comprehensive statistical evaluation protocol—incorporating large-scale repeated runs and confidence interval analyses—to validate its robustness using realistic, highly dynamic vehicular datasets.

## Figures and Tables

**Table 1:** Qualitative comparison with representative IoV authentication and FL security studies.

Scheme	Blockchain-BackedAuthentication	FL SecuritySupport	Trust/ReputationMechanism	Cross-LayerCoupling	BatchAuthentication	Model-PoisoningDefense	Main Limitation
TABMA [7]	Yes	No	Yes	No	Yes	No	Comm.-layer only; no FL trust control
BATS [17]	Yes	No	Yes	No	No	No	Auth./trust anchoring only
BEBA [18]	Yes	No	Limited	No	Yes	No	No FL-aware trust coupling
DAG-FL [8]	No	Yes	Yes	Partial	No	Yes	No source identity authentication
EBCFL [23]	No	Yes	Limited	Partial	No	Partial	FL orchestration focused; weak comm. security
MARL-FL [24]	No	Yes	Yes	Partial	No	Yes	No blockchain-backed identity protection
TrustBlockFL [25]	Yes	Yes	Yes	Partial	No	Yes	Lacks strict cross-layer cryptographic reuse
DAG-CTFL	Yes	Yes	Yes	Yes	Yes	Yes	Higher design complexity

**Table 2:** Summary of main notations.

Symbol	Description
TA	Trusted authority for initialization and partial-key generation.
$V_{i}$, $RSU_{j}$, $BS_{k}$	Vehicle, roadside unit, and base-station node.
$PID_{i}$	Pseudonymous identity of vehicle $V_{i}$.
$p,q,E(F_{p}),P$	ECC field, group order, curve, and base point.
$s,P_{pub}$	Master secret key and master public key.
$ID_{i},UID_{i},PW_{i}$	Real identity, user identity, and password.
$A_{i},B_{i},C_{i}$	Registration/authentication values.
$x_{i},X_{i}$	Vehicle secret and corresponding public point.
$M_{i},M_{i}^{crit},t_{i},\Delta T$	Routine message, critical message, timestamp, and freshness threshold.
$\sigma_{i}^{ecc},\sigma_{i}^{pq},\Sigma_{hyb}$	ECC, PQ, and hybrid signatures.
$V_{ecc},V_{pq},V_{hyb}$	ECC, PQ, and hybrid verification functions.
$M_{crit}$	Critical-message set requiring PQ protection.
$DT_{s}\left(i\right),IT_{s}\left(i\right),T_{s}\left(i\right),T_{s}^{new}\left(i\right)$	Direct, indirect, fused, and updated trust values.
$T_{init},T_{min},\gamma$	Initial trust, admission threshold, and penalty coefficient.
$F_{s}\left(i\right),P_{s}\left(i\right),\theta$	Behavior score, penalty term, and time-decay factor.
$\Delta w_{i},\Delta w_{i}^{\prime },\Delta w_{RSU},\Delta w_{global}$	Local, noised, RSU-level, and global model updates.
$w_{g},w_{g}^{new}$	Current and updated global model parameters.
ϵ,σ	DP budget and Gaussian noise scale.
$h_{1},\ldots ,h_{5},H\left(\cdot \right)$	Hash functions used in authentication and PQ signing.
$Adv_{hyb}^{EUF-CMA}$	Hybrid-signature forgery advantage.

**Table 3:** ProVerif formal security verification results.

Security Attribute	Verification Query	Result	Conclusion
Anti-Forgery	inj-event(messageVerified) ==> inj-event(beginSign) && inj-event(messageSent)	true	Resist forgery attacks
Anti-Tampering	event(messageVerified) ==> event(beginSign) && event(messageSent)	true	Resist tampering attacks
Anti-Replay	inj-event(messageVerified) ==> inj-event(messageSent)	true	Resist replay attacks
Privacy Preservation	not attacker(ID) && not attacker($x_{i}$)	true	Identity privacy protected

**Table 4:** Comparison of cryptographic operations for signing and verification.

Scheme	Signing Operation	Verification Operation	Total Core Operations
EIDA [15]	3 ECC Scalar Mul	3 ECC Scalar Mul + 2 ECC Point Add	6 Scalar Mul + 2 Point Add
CASA [16]	3 ECC Scalar Mul	3 ECC Scalar Mul	6 Scalar Mul
RSMA [31]	1 ECC Scalar Mul	2 ECC Scalar Mul + 2 ECC Point Add	3 Scalar Mul + 2 Point Add
TABMA [7]	1 ECC Scalar Mul	2 ECC Scalar Mul + 2 ECC Point Add	3 Scalar Mul + 2 Point Add
DAG-CTFL (Ours)	1 ECC Scalar Mul	2 ECC Scalar Mul + 2 ECC Point Add	3 Scalar Mul + 2 Point Add

**Table 5:** Final model accuracy comparison in benign scenarios (mean ± std., %).

Dataset	Model	FedAvg	DSSFL [33]	DP-FedAvg [34]	DAG-FL [8]	DAG-CTFL (Ours)
MNIST	MLP	98.92±0.18	98.76±0.21	96.34±0.43	97.80±0.29	97.90±0.25
	CNN	99.91±0.04	99.87±0.05	98.25±0.31	99.50±0.09	99.40±0.11
CIFAR-10	MLP	60.82±0.74	60.59±0.81	55.12±1.03	60.59±0.70	63.50±0.68
	CNN	71.03±0.61	70.85±0.66	55.37±1.12	70.59±0.59	70.80±0.57

**Table 6:** Model accuracy comparison under different malicious node ratios (MNIST-MLP, mean ± std., %).

Malicious Ratio	FedAvg	DSSFL [33]	DP-FedAvg [34]	DAG-FL [8]	DAG-CTFL (Ours)
0%	98.92±0.18	98.76±0.21	96.34±0.43	97.80±0.29	97.90±0.25
5%	68.35±1.82	56.72±2.10	42.68±2.45	87.63±0.73	87.81±0.69
10%	42.29±2.54	43.81±2.31	22.57±2.84	86.95±0.81	87.12±0.72
15%	27.71±2.87	34.95±2.62	13.74±2.51	86.43±0.86	86.72±0.64
20%	18.35±2.43	22.17±2.38	8.92±1.76	81.76±1.02	82.35±0.94

**Table 7:** Average message utilization under different malicious node ratios (mean ± std.).

Malicious Ratio	EIDA [15]	TABMA [7]	DAG-CTFL (Ours)
10%	0.303±0.015	0.625±0.014	0.645±0.011
20%	0.208±0.013	0.542±0.016	0.565±0.012
30%	0.176±0.012	0.501±0.018	0.526±0.014

## Data Availability

Data are contained within the article.
